# Improvement of Total Flavonoids from *Dracocephalum moldavica* L. in Rats with Chronic Mountain Sickness through ^1^H-NMR Metabonomics

**DOI:** 10.1155/2021/6695346

**Published:** 2021-05-03

**Authors:** Atiguli Maimaiti, Yang Tao, Wang Minmin, Miao Weiwei, Shi Wenhui, Ainiwaer Aikemu, Dilinuer Maimaitiyiming

**Affiliations:** ^1^Heart Center, The First Affiliated Hospital of Xinjiang Medical University, Urumqi 830054, China; ^2^Central Laboratory, Xinjiang Medical University, Urumqi 830054, China; ^3^College of Pharmacy, Xinjiang Medical University, Urumqi 830054, China; ^4^Pharmacology Department, Sanquan College of Xinxiang Medical University, Xinxiang 453000, China; ^5^Key Laboratory of Special Environmental Medicine of Xinjiang, General Hospital of Xinjiang Military Region of PLA, Urumqi 830054, China

## Abstract

**Background:**

We analyzed the effects of total flavonoids from *Dracocephalum moldavica L*. (*D. moldavica* L.) on improving chronic mountain sickness (CMS) in rats using the NMR hydrogen spectrum (^1^H-NMR) metabonomics technology.

**Method:**

We extracted the total flavonoids of *D. moldavica L* with 60% ethanol reflux. A CMS model was established with 48 Sprague–Dawley (SD) rats, which were then randomly divided into six groups (*n* = 8): control group (normal saline, 0.4 mL/100 g/d, ig); model group (normal saline, 0.4 mL/100 g/d, ig); nifedipine group (nifedipine tablets, 2.7 mg/kg/d, ig); and high-, middle-, and low-dose groups of total flavonoids from *D. moldavica L*. (DML.H, DML.M, and DML.L, receiving total flavonoids from *D. moldavica* L. at 400, 200, and 100 mg/kg/d, ig, respectively). The sera of the rats in all the groups were determined, and NMR hydrogen spectrum metabolomics was analyzed. The serum contents of apolipoproteins A1 (Apo-A1) and *E* (Apo-E) were determined, and histopathological changes in the brain tissue of each group were observed.

**Results:**

Serum tests showed that total flavonoids from *D. moldavica* L. significantly increased the Apo-A1 and Apo-E levels in rats with CMS (*P* < 0.05). The results of serum metabonomics showed that total flavonoids from *D. moldavica L* can alleviate amino acid, energy, and lipid metabolism disorders in rats with CMS. Pathohistological examination of brain tissue showed that these flavonoids improved pathological changes, such as meningeal vasodilation, hyperemia, edema of brain parenchyma, inflammatory cell infiltration, increase in perivascular space, and increase in pyramidal cells.

**Conclusion:**

Total flavonoids from *D. moldavica* L. have potential therapeutic effects on CMS. The possible mechanism is the reduction of oxidative damage through the alleviation of metabolism disorder.

## 1. Introduction

Chronic mountain sickness (CMS), also called Monge's disease, refers to a kind of comprehensive high-altitude disease that develops in people living in places above 2500 m altitude for a long time. CMS includes high-altitude polycythemia, heart disease, and pulmonary hypertension [1]. The possible pathogenesis involves changes in energy metabolism pathways due to systemic hypoxia, excessive red blood cell proliferation, chronic inflammation, and oxidative stress response [2]. Currently, the most effective way of alleviating CMS is moving an affected individual to a low-altitude place. However, this approach is greatly restricted by economic and social factors. Other methods include reducing pulmonary hypertension, improving hypoxia, reducing hemoglobin (Hb) level, and controlling the infection.

Traditional Chinese medicine has a long history of use in clinical treatment, with the characteristics of multiple ingredients, multiple targets, and multiple pathways, which may be an effective source for new drug development. *D. moldavica* L. is an annual plant belonging to the family Labiatae. This herbaceous annual plant is widely found in different regions, including China, Russia, Siberia, Eastern Europe, and Central Europe. Its dry part of the ground is made into dripping pills, which have the effects of replenishing the heart and brain, promoting blood circulation, reducing phlegm, diuresis and relieving cough, relieving pain, and promoting detoxification; therefore, it is commonly used clinically to treat coronary heart disease, hypertension, angina pectoris, arteriosclerosis, myocardial ischemia, and other diseases [3].

Previous research found that the extract of *D. moldavica L* (water extract, ethanol extract) improved CMS by reducing pulmonary artery pressure, reducing oxidative stress, and favoring myocardial protection [4, 5]. However, there are many ingredients extracted from *D. moldavica L*, and it is necessary to continue studying the biological activity of its components. Flavonoids from *D. moldavica* L. have a wide range of biological activities, such as lowering blood pressure and protecting against cerebral ischemia [6]. Therefore, we used total flavonoids from *D. moldavica* L. to treat rats with CMS. The results showed that total flavonoids from *D. moldavica* L. had a certain therapeutic effect on rats with chronic altitude sickness, specifically the effect manifested by the reduction of pulmonary artery pressure and organ protection [7]. However, the exact mechanism of action requires further study.

In this study, a CMS rat model was established by simulating a 5000 m high plateau environment. After intervention with different doses of total flavonoids from *D. moldavica* L., serum metabolites before and after the administration of the flavonoids were analyzed using NMR hydrogen spectrum (^1^H-NMR) metabonomics. The serum contents of apolipoproteins A1 (Apo-A1) and *E* (Apo-E) were determined, and histopathological changes in the brain tissue of each group of rats were examined. Through Metabolomics Pathway Analysis (MetPA), we explored the mechanism of the total flavonoids from *D. moldavica* L. in improving CMS in rats from the perspective of systems biology.

## 2. Methods

### 2.1. Animals

A total of 48 SD rats (24 males and 24 females, 200 ± 30 g) were used. The rats were bought from Experimental Animal Center, Xinjiang Medical University. The animal license number was SCXK (Xin) 2016–0003.

### 2.2. Instruments

We used the following instruments: nuclear magnetic resonance spectrometer (Type: Inova 600, Varian Company, USA); low-temperature ultracentrifuge (Beckman Company, USA); DW-86L338 ultralow-temperature freezer (Haier Corporation, China); Thermo Scientific Plate Washer (AC8; Thermo Lab Systems, Finland); electrophysiological recorder (BL-420, Chengdu Techman Company, China); small animal ventilator (ks606731, Beijing Kesijia Company, China); northwest special environment artificial experimental cabin (located in Xinjiang Key Laboratory).

### 2.3. Reagents and Drugs

We used the following reagents and drugs: *D. moldavica L*. (Decoction Factory, Xinjiang Madison Pharmaceutical Co., Ltd, Batch No. M30062307; the quality inspection report provided by Xinjiang Maidisen Pharmaceutical Company was identified as the dry part of ground from *Dracocephalum moldavica* Linn. by Miregiuli, the quality inspector of Madison); nifedipine tablets (Shanxi Yun Peng Pharmaceutical Co., Ltd, Batch No. F160601); rat Apo-A1 kit (Batch No. AD20180708, Andy Gene Biotechnology Co., Ltd., China); rat Apo-E ELISA kit (Batch No. JL11304, Shanghai Jianglai Biological Technology Co., Ltd.); heavy water (Cambridge Isotope Laboratories, Inc.); NaCl, K_2_HPO_4_, and NaH_2_PO_4_ (Tianjin Guangfu Fine Chemical Research Institute, analytical purity); nuclear magnetic tube; distilled water.

### 2.4. Extraction of Total Flavonoids from *D. moldavica L*

We took 1 kg of *D. moldavica L* powder and extracted it with 60% ethanol aqueous solution reflux three times. The reflux extraction temperature was 60°C, and the reflux extraction time was 2 h. The amount of ethanol aqueous solution during the reflux extraction was 30 times higher than the weight of the *D. moldavica L* powder. The extract was obtained after reflux extraction, and it was concentrated to 10 times the weight of *D. moldavica L* powder to obtain the first concentrated solution; then, absolute ethanol was added to the first concentrated solution to obtain a mixed solution. The mass percentage was 70%, and the product was kept at 4°C for 24 h and then filtered. The filtrate was concentrated to 10 times the weight of the powdered herbs to obtain the second concentrated solution; the concentrated solution was adsorbed by HPD600 macroporous resin. After adsorption, the adsorption residue was discarded, and the macroporous resin was eluted with four column volumes of deionized water. After washing with water, the washing solution was discarded; then, we used five column volumes of aqueous ethanol solution (5%, v/v) for the first elution of ethanol. After the elution, the first eluate of ethanol was discarded, and then 5 times the column volume was used for the second elution of ethanol with 30% by volume. The volume percentage of the column volume was 60% aqueous ethanol solution for three times the ethanol elution. We collected the second and the third eluates of ethanol; these two eluates were mixed, concentrated to a thick paste with a relative density of 1.20 at 20°C, and then dried at 60°C for 8 h. After drying, the *D. moldavica L* extract was obtained. The mass percentages of luteolin-7-O-*β*-D-glucuronide, apigenin-7-O-*β*-D-glucuronide, rosmarinic acid, geraniol-7-O-*β*-D-glucuronide, tianthisin, and robinin-7-O-*β*-D-glucuronide were 7.5%, 1.7%, 9.1%, 5.7%, 14.7%, and 11.5%, respectively; the mass percentage of total flavonoids was 51.5%.

### 2.5. Establishment of Chronic Mountain Sickness Model

Eight rats were randomly selected and fed in a plain environment (altitude, 720 m; temperature, 18–26°C; humidity, 40%–60%; atmospheric pressure, 93.2 kPa; partial pressure of oxygen, 19.54 kPa) for 30 days. They had free access to food and water. The remaining 40 rats were placed in a hypobaric oxygen chamber (simulated altitude, 5000 m; temperature, 18–26°C; humidity, 40%–60%; atmospheric pressure, 54.1 kPa; partial pressure of oxygen, 10.84 kPa) for 30 days to establish a CMS model. They had free access to food and water.

### 2.6. Grouping and Administration Method

A total of 40 CMS rats were randomly divided into the model group (MG, normal saline 0.4 mL/100 g once a day, intragastric administration for 15 days), nifedipine group (NE, nifedipine tablets, 2.7 mg/kg once a day, intragastric administration for 15 days), high-dose group (DML-H, total flavonoids from *D. moldavica L*. 400 mg/kg once a day, intragastric administration for 15 days), middle-dose group (DML-M, 200 mg/kg once a day, intragastric administration for 15 days), and low-dose group (DML-L, 100 mg/kg once a day, intragastric administration for 15 days). Eight rats in the plain environment were the control group (CG, normal saline, 0.4 mL/100 g once a day, intragastric administration for 15 days).

The experimental design and implementation were approved by the Animal Ethics Committee of the First Affiliated Hospital of Xinjiang Medical University (approval number: IACUC-20160218009). Each rat in each group was anesthetized with 3% pentobarbital sodium by intraperitoneal injection. At the end of the experiments, the rats were sacrificed by cervical dislocation.

### 2.7. Determination of Pulmonary Artery Pressure in Rats

At 24 h after drug administration, each rat in each group was anesthetized with 3% pentobarbital sodium by intraperitoneal injection and fixed on an operating table in a supine state. The chest was opened, and the lungs and heart were fully exposed. The jugular vein was stripped out, and a tube was inserted into the pulmonary artery via the right atrium and right ventricle. Pulmonary artery pressure was recorded.

### 2.8. Determination of Serum Apo-A1 and Apo-E Contents in rats in Different Groups

After the determination of pulmonary artery pressure, blood was collected from the abdominal aorta; it was placed at room temperature for 30 min and centrifuged at 3000 r/min for 20 min at a low temperature. Then, the supernatant was collected and stored in a refrigerator at −80°C. The determination method provided in the kit's instructions was adopted to determine the serum Apo-A1 and Apo-E contents.

### 2.9. Determination of Blood Nuclear Magnetic Resonance Spectrum of Rats in Different Groups

The serum samples (200 *µ*L each) were collected, and 400 *µ*L of phosphoric acid buffer prepared with heavy water was added to each sample. The samples were allowed to settle at room temperature for 10 min and then centrifuged at 10 000 r/min for 10 min. Approximately 550 *µ*L of each supernatant was poured into a 5 mm NMR tube. An Inova600 nuclear magnetic resonance spectrometer was used in determining the hydrogen spectrum by NOESY-PRESAT 1D (RD-90-t1-90-tm-90-ACQ) pulse sequence. The 1H-NMR frequency was 599.13 MHz. Accumulative scanning was performed 64 times. A total of 32,768 sampling data points were obtained, and the spectral width was 20 mg/L. The sampling delay was 2 s; the time of each scan was 1.64 s; testing temperature was 25°C. A presaturation method was used to suppress the water peak.

### 2.10. ^1^H-NMR Map Processing and Multivariate Statistical Analysis

^1^H-NMR map phase and baseline were adjusted using MestReNova 12 (Mestrelab Research, Santiago de Compostella, Spain). On the basis of the proton signal with the 5.233 ppm chemical shift of *a*-glucose, we corrected the chemical shift, phase, and baseline. The integral of 0.03 ppm equal-width splits was carried out in the map of 9.00–0.5 ppm. The water peak at 4.5–5.0 ppm was cut off. After the normalization of residual integral data, multivariate statistical analysis was carried out using SIMCA-p 14.0 (Umetrics, Sweden). Principle component analysis (PCA) was used to reflect inherent differences and similarities among samples and show the original classification status of data. Furthermore, partial least-squares discriminant analysis (PLS-DA) and orthogonal partial least-squares discriminant analysis (OPLS-DA) were carried out. The effectiveness of PLS-DA was verified by permutation tests (200 times). With the VIP value of OPLS-DA and the corresponding s-plot, different metabolites between groups were identified. Finally, information on the metabolites was imported to the pathway analysis module of MetaboAnalyst 4.0 (https://www.metaboanalyst.ca). In this module, pathway enrichment analysis was combined with pathway topology analysis for the screening of key metabolic pathways.

### 2.11. Pathological Examination of Brain Tissue of Rats in Each Group

At 24 h after the last administration, the animals were euthanized. Brain tissues were extracted and cleaned with normal saline. The tissues were then fixed in 10% formalin solution, dehydrated by gradient ethanol, vitrified by dimethylbenzene, embedded in paraffin, and stained with hematoxylin and eosin. The tissues were cut into pathological sections, and morphological changes in the cells and tissues were observed under an optical microscope.

### 2.12. Statistical Analysis

The serological indexes of the rats in each group were analyzed with SPSS22.0. Normality tests and homogeneity of variance tests were carried out. Normally distributed data were presented as mean and standard deviation. The mean values between the groups were compared by one-way ANOVA. When the mean values between the groups were different, pairwise comparisons were performed through LSD, SNK, and other methods. The significance level was set at 0.05.

## 3. Results

### 3.1. Effects of Total Flavonoids from *D. moldavica* L. on Pulmonary Artery Pressure

Compared with the rats in the CG, the rats in MG, NE, DML-H, DML-M, and DML-L groups showed increased pulmonary artery pressure (*P* < 0.05). Compared with the rats in MG, those in NE, DML-H, DML-M, and DML-L groups had decreased pulmonary artery pressure (*P* < 0.05). Compared with the rats in the NE group, those in DML-H and DML-M groups showed decreased pulmonary artery pressure (*P* < 0.05; see Table 1).

### 3.2. Identification and Analysis of ^1^H-NMR Spectra

**Figure 1:**
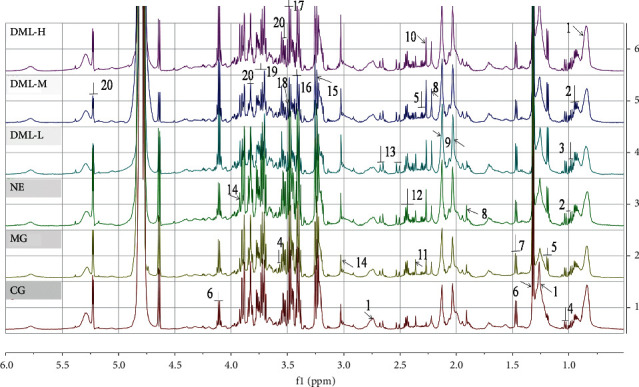
the ^1^H-NMR spectra of the sera of the rats in each group.

The ^1^H-NMR spectra of the sera of the rats in each group are shown in Figure 1. Based on chemical shift, coupling constant, and peak nature, BMRB (Biological Magnetic Resonance Data Bank) was used to identify the spectra. A total of 21 metabolites in the rat sera were identified, including amino acids, sugars, and fats. The results are shown in Table 2. The labels in Figure 1 correspond to those in Table 2.

**Figure 2:**
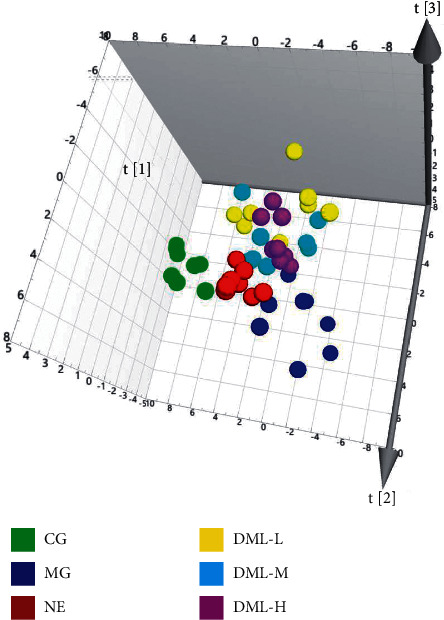
Multiple statistical analysis from the ^1^H-NMR spectra of rat serum in CG and MG.

The results of OPLS-DA analysis performed on the sera of rats in the CMS model group are shown in Figure 2. As shown in Figure 2-A, the samples of the normal and model groups were separated along the *t* [1] axis, indicating that the CMS model was copied successfully. By establishing an S-plots map corresponding to the OPLS-DA model (Figure 2-B), the VIP value (VIP > 1) was combined to screen out. An independent-sample *t*-test was carried out for the peak area of the screened metabolites, and potential biomarkers were identified.

Nineteen potential biomarkers obtained from the sera included upregulated or downregulated lipids (LDL/VLDL), isoleucine, leucine, valine, 3-hydroxybutyrate, lactate, alanine, acetate, glycoprotein, acetoacetate, pyruvate, glutamine, citrate, creatine, taurine, proline, glycine, citrulline, and alpha-glucose.

**Figure 3:**
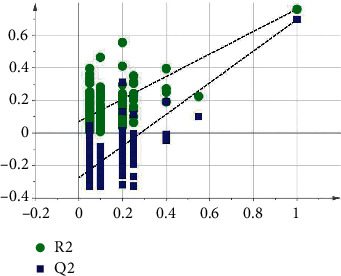
Permutation test.

According to the OPLS-DA model arrangement, *R*^2^ and Q^2^ values from any random arrangement on the left in the experiment were smaller than the original values on the right, indicating that the prediction ability of the original model was greater than that of any randomly arranged *y* variable; that is, the model was effective, and the subsequent analysis was available. The verification diagram obtained is shown in Figure 3, and the differential metabolites in sera of the normal and model groups are listed in Table 3.

Multivariate statistical analysis of ^1^H-NMR spectra in sera of rats in each group with total flavonoids from *D. moldavica L*.

**Figure 4:**
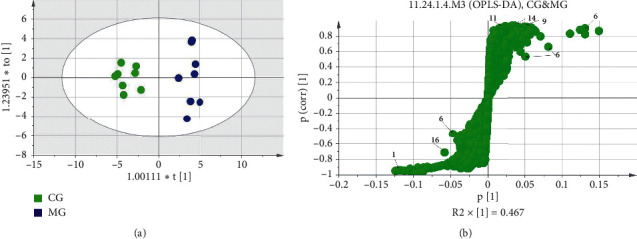
Multiple statistical analysis from ^1^H-NMR spectra of rat serum.

**Figure 5:**
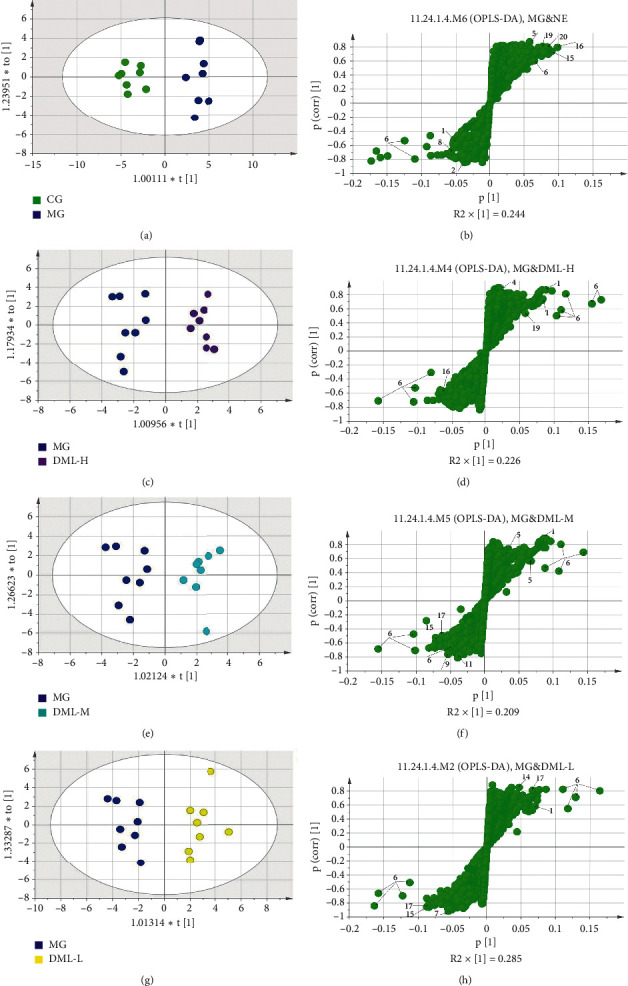
PLS-DA score pots of rats in each group.

Sera of each administration group were analyzed using PLS-DA, and the results are shown in Figure 4. The space that each group occupied was basically independent, indicating differences among the groups. OPLS-DA was used to compare sera of each administration group with those of the model group; the results are shown in Figure 5. The samples of each administration group and those of the model group were separated along the *t* [1] axis (Figures 5(a), 5(c), 5(e), and 5(g)). The S-plots diagram showed the change in the related metabolites (Figures 5(b), 5(d), 5(f), and 5(h)). Table 3 lists the metabolites with a significant difference in the integral area compared with the model group; these metabolites are involved in amino acid metabolism, lipid metabolism, and glycolysis.

### 3.3. Differential Metabolite Pathway Analysis

**Figure 6:**
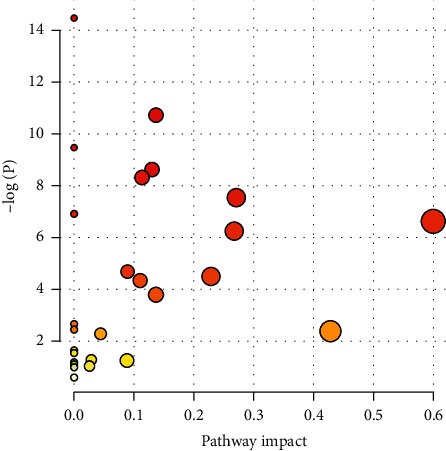
Summary diagram of pathway analysis with MetPA.

The differential metabolites were imported in the MetaboAnalyst (http://www.metaboanalyst.ca/) pathway analysis platform. The results are shown in Figure 6. A pathway impact represents the importance of the metabolic pathway obtained by the topological analysis, and -lg *P* represents the significance level of the metabolic pathway enrichment analysis. Greater pathway impact and -lg *P* of a metabolic pathway indicate a higher difference in metabolism among the groups. In this study, the metabolic pathway impact threshold was set at 0.10. Pathways with an impact value higher than 0.10 were regarded as the potential target metabolic pathways. They included 10 metabolic pathways, such as glyoxylate and dicarboxylate metabolism; glycolysis/gluconeogenesis, alanine, aspartate, and glutamate metabolism; glycine, serine, and threonine metabolism; synthesis and degradation of ketone bodies; pyruvate metabolism; arginine biosynthesis; butanoate metabolism; citrate cycle (TCA cycle) and taurine and hypotaurine metabolism (Table 4).

Effects of total flavonoids from *D. moldavica* L. on Apo-A1 and Apo-E contents in the sera of rats in each group.

As shown in Table 5, serum Apo-A1 and Apo-E contents in the MG, NE, DML-M, and DML-L rats were lower than those in the CG rats, and the differences were statistically significant (*P* < 0.05). Serum Apo-A1 and Apo-E contents in the NE, DML-H, DML-M, and DML-L rats were significantly higher than those in the MG rats (*P* < 0.05). Compared with NE rats, serum Apo-A1 and Apo-E contents were significantly higher in DML-H rats and significantly lower in DML-M and DML-L rats (*P* < 0.05).

### 3.4. Effects of Total Flavonoids from *D. moldavica* L. as Shown in Pathological Examination of Brain Tissue

**Figure 7:**
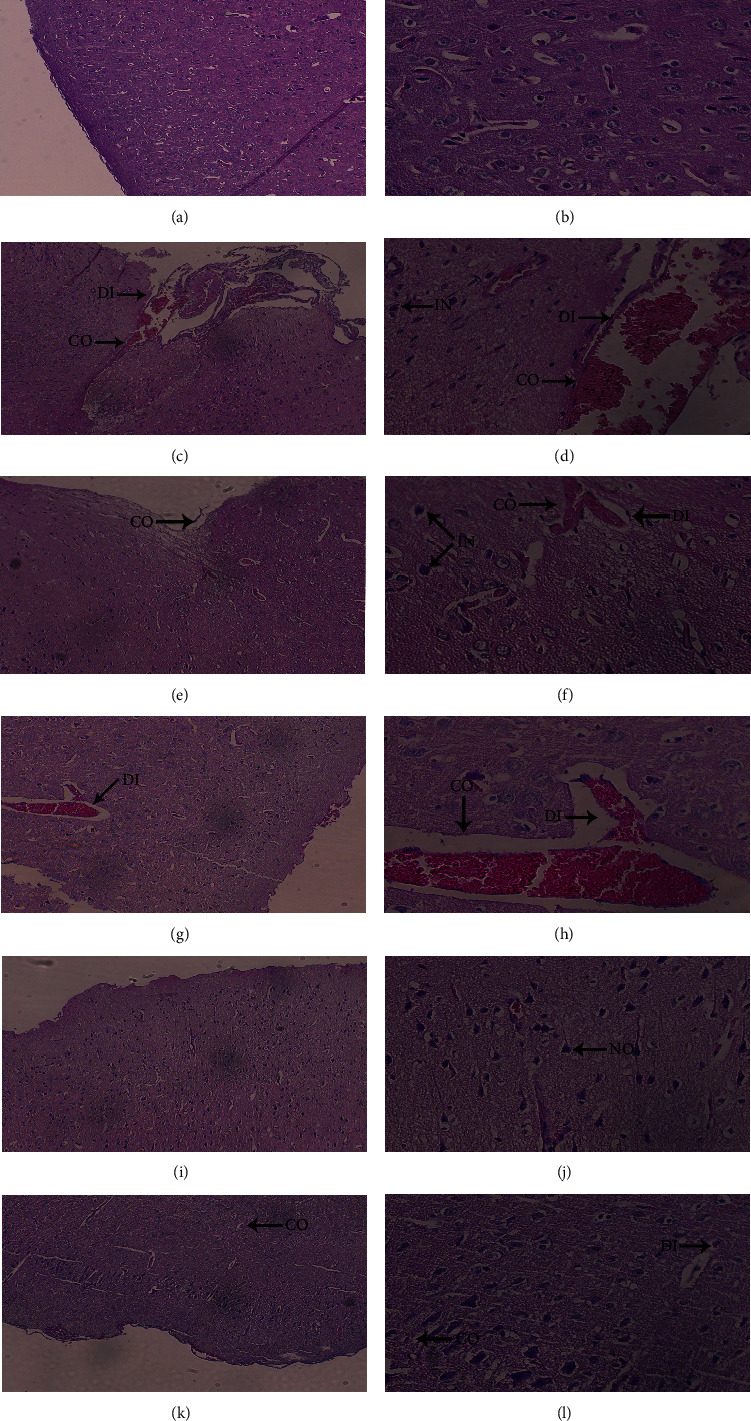
Micrographs of pulmonary artery pathology of rats in each goup (HE staining).

Thin meninges were observed under low-power (Figure 7(a)) and high-power magnification (Figure 7(b)) in the samples of CG rats. No abnormal exudate was found in the meninges. In the parenchyma of the cerebral cortex, pyramidal cells were normal in size; no abnormal lesions, such as dilation, hemorrhage, hyperemia, and edema, were found in blood vessels.

Under low-power magnification (Figure 7(c)), the meningeal vessels in the brain tissue of the MG rats were dilated and congested, and perivascular cavities of the brain parenchyma were dilated with exudate. Under high-power magnification (Figure 7(d)), blood vessels of the brain parenchyma showed vasodilation, hemorrhage, and edema, which were accompanied by inflammatory cell infiltration. The pyramidal cells size of the brain increased.

Under low-power magnification (Figure 7(e)), hyperemia was obvious in the meninges of the NE rats. Under high-power magnification (Figure 7(f)), blood vessels in the cerebral cortex were dilated and congested, and pyramidal cells were deformed. No obvious infiltration by inflammatory cells was observed.

Under low-power magnification (Figure 7(g)), mild hemorrhage was observed in the meninges of the DML-L rats. Under high-power magnification (Figure 7(h)), vasodilation, hemorrhage, and inflammatory cell infiltration were observed in the brain interstitium, and the parenchyma was loose near the hemorrhage.

Under low-power magnification (Figure 7(i)), no obvious abnormality was found in the meninges of the DML-M rats, and neurons in the cerebral cortex were evenly distributed. Under high-power magnification (Figure 7(j)), the pyramidal cells were normal in size, and no obvious abnormalities were found in the cerebral parenchymal vessels.

Under low-power magnification (Figure 7(k)), mild hemorrhage was observed in the meninges of the DML-H rats. Under high-power magnification (Figure 7(l)), the meninges were thick, some blood vessels in the granular layer outside the cerebral cortex were slightly bleeding, and mild edema was observed near the hemorrhage.

## 4. Discussion

In this article, ^1^H-NMR metabonomics technology was used to analyze differential metabolites in serum, including those involved in glyoxylate and dicarboxylate metabolism; glycolysis/gluconeogenesis, alanine, aspartate, and glutamate metabolism; glycine, serine, and threonine metabolism; synthesis and degradation of ketone bodies; pyruvate metabolism; arginine biosynthesis; butanoate metabolism; tricarboxylic acid cycle (TCA cycle); taurine and hypotaurine metabolism. Ten metabolic pathways were included in total. The mechanism of action of total flavonoids from *D. moldavica* L. on CMS is mainly related to carbohydrate, lipid, and amino acid metabolism, as well as to anti-inflammatory and antioxidative effects.

### 4.1. Energy Metabolism

In a high-altitude environment, cellular aerobic metabolism cannot meet the demand for energy owing to insufficient oxygen supply in cells/mitochondria, hypoxic injury in cells, and an increase in the basal metabolic rate [8]. Nemkov et al. [9]^.^ reported that under hypoxia conditions, the proportion of pyruvate/lactate, represented by nicotinamide adenine dinucleotide/nicotinamide adenine dinucleotide (NADH/NAD+) ratio, increased and the content of oxidized glutathione decreased in red blood cells. Our findings showed that the serum contents of lactate and pyruvate in the CMS model group were upregulated compared with those in the control group, indicating that anaerobic glycolysis was strengthened. In hypoxia, hypoxia-inducible factor-*α* (HIF-*α*) is not hydroxylated but accumulates in the nuclei, thereby changing the metabolism pathway from oxidative phosphorylation to anaerobic glycolysis. In glycolysis, pyruvate oxidation is inhibited, and thus it cannot enter the citric acid cycle smoothly; pyruvate is finally reduced to lactate, resulting in the accumulation of lactate and pyruvate [10]. HIF acts on the promoter of phosphoenolpyruvate kinase to activate the Cori cycle and promote lactate gluconeogenesis [11]; lactic acid can be reused through gluconeogenesis, whereas lactate beyond the body's processing capacity alters the acid–base balance in the internal environment. In glycolysis, energy production is fast, but the utilization of glucose is low. By consuming 1 mole of glucose, only two molecules of adenosine triphosphate (ATP) are obtained. Therefore, the dependence of highly regulated HIF on glucose uptake is strengthened to produce enough ATP [12]. Mature erythrocytes are the main cells that actively participate in energy metabolism. The molecular mechanism involved in HIF-induced erythropoiesis may be related to the energy metabolism pathway in a high-altitude hypobaric hypoxia environment. Our results showed that the contents of multiple amino acids and *a*-glucose in the blood of the CMS rats increased, indicating that amino acid gluconeogenesis was strengthened to supplement blood glucose and maintain glucose-dependent energy production during glycolysis. After the gavage administration of different doses of total flavonoids from *D. moldavica* L., the contents of lactate and pyruvate significantly decreased and the level of *a*-glucose decreased in the blood of the CMS rats. This result indicates that the total flavonoids from *D. moldavica* L. can improve energy metabolism, weaken anaerobic glycolysis, and enhance hypoxia tolerance. Geraniol-7-O-*β*-D-glucuronide contained in the total flavonoids of *D. moldavica L* can inhibit glycolysis and citric acid cycle and increase the level of ATP. Therefore, we speculate that the improved body's energy metabolism by the total flavonoids of *D. moldavica L* may be related to geraniol-7-O-*β*-D-glucuronide.

### 4.2. Amino Acid Metabolism

In a high-altitude hypoxia environment, changes in energy metabolism, glycolysis, and gluconeogenesis were observed in the CMS rats. Dilinuer et al. performed metabonomics analysis after establishing a high mountain sickness rat model. They found that the levels of tyrosine, leucine, phenylalanine, and other amino acids changed in the serum of high-altitude rats [13]. In the present study, the serum contents of several amino acids (isoleucine, leucine, valine, alanine, taurine, proline, and glycine) were higher in the CMS rats than those in the rats in the plain group. In addition to gluconeogenesis, amino acids also play other physiological roles. Leucine, isoleucine, and valine are common branched-chain amino acids that can improve glucose metabolism and the inflammatory response of adipose tissue in rats [14, 15]. Insulin resistance is another pathological change of the endocrine system in high mountain sickness. Branched-chain amino acids can stimulate insulin secretion, enhance glucose absorption in peripheral blood by muscles, increase the production capacity, and indirectly alleviate insulin resistance, which is conducive to energy supply. The contents of branched-chain amino acids in the rats of the CMS model group were significantly higher than those in the rats of the plain group. The increase in amino acid content with anti-inflammatory effect explains the pathological characteristics of CMS from the side and shows that the effect of total flavonoids from *D.moldavica* L. on the metabolism of amino acid promotes glucose utilization and energy metabolism. After the DML administration, the serum contents of various amino acids (isoleucine, valine, alanine, taurine, proline, and glycine) significantly decreased. Possible reasons include the anti-inflammatory and antioxidative effects of the total flavonoids from *D. moldavica* L. and the decrease in the body's demand for its own anti-inflammatory substances; however, its specific mechanism needs to be confirmed by further experiments. In addition, taurine can inhibit the apoptosis rate of neuroglia cells in the early and middle-late stages induced by hypoxia [16]. Excitatory and inhibitory neurotransmitters play a key role in the induction and adaptation of central and peripheral nervous systems to a hypoxic environment. Under normal conditions, glycine is an inhibitory neural transmitter, acts through a pathway similar to *γ*-aminobutyric acid (GABA), and has a synergistic effect with N-Methyl-D-aspartic acid (NMDA) receptors. By changing the pathway of NMDA receptors, it can significantly enhance the effects of glutamate and other positive amino acids. *Glycine*, which inhibits the long-term sustained excitation of nerve cells in the hippocampus and brain stem, the main negative neuromessenger [17]. By detecting glycine in rat brains, Liu et al. found that the levels of amino acids in the entire brain increased significantly with the improvement of hypoxia tolerance; GABA-like effects enhanced hypoxia tolerance [18]. Serum glycine content in the rats in the high-altitude model group significantly increased. According to previous studies, a high-altitude environment increases excitability by stimulating neurons and inhibits the sustained overexcitation of the central nervous system by increasing glycine content. After the administration of total flavonoids from *D. moldavica* L., serum glycine content in the rats in the high-altitude model group significantly decreased. This result indicates that these flavonoids protect the central nervous system by affecting neurotransmitters in the brain under hypoxic environments.

### 4.3. Lipid Metabolism

Lipids store energy in organisms. When the capacity of glycolysis cannot maintain the normal energy supply of the body, the mobilization and decomposition of fat produce ATP for use. Under nonhypoxic conditions, fatty acids undergo repeated *ß* oxidation to generate acetyl coenzyme A (acetyl CoA), which participates in the tricarboxylic acid cycle (TCAC). In hypoxia, the oxidation of fatty acids is downregulated [8], and acetyl CoA produced from *ß* oxidation cannot be completely oxidized by TCAC to acetoacetic acid. Acetoacetic acid is further decomposed into acetone, *ß* hydroxybutyric acid, and other ketones. In this article, we showed that serum acetoacetic acid in the CMS model group was significantly higher than that in the plain group, indicating that the capacity of fat mobilization and decomposition can be enhanced to meet the energy demand of the body in a high-altitude environment. Ketone bodies are intermediate products of fatty acid decomposition and a method of the energy output of the liver. Under the conditions of energy shortage due to oxygen deficiency at high altitudes, the heart, brain, kidneys, and skeletal muscles can use ketone bodies to produce energy. However, if the production of ketone bodies is larger than the scope of organism treatment, the process causes the accumulation of ketone bodies and, in serious cases, may even result in ketosis. After the administration of the total flavonoids from *D. moldavica* L., acetoacetic acid content significantly decreased in the CMS model rats. This result indicates that flavonoids from *D. moldavica* L. can improve lipid oxidative metabolism in CMS rats. The possible mechanisms include increasing the effective utilization of sugar and reducing the demand for lipid energy supply. These mechanisms weaken fat mobilization and reduce the sources of ketone production. However, this theory needs to be confirmed by strong experimental evidence; it can be confirmed by determining muscle and fat contents and the level and activity of various speed-limiting enzymes in the ketone capitalized process and by performing liver biopsy in CMS rats.

Apolipoproteins are mainly responsible for transferring lipids to the whole body. Apo-E is mainly produced in the liver and brain. In the brain, astrocytes are responsible for its production and release. Apo-E is the most critical apolipoprotein in the central nervous system and the main lipid transport carrier in the cerebrospinal fluid. Apo-E exerts a protective effect on astrocytes [19, 20]. In the present study, Apo-E was detected in sera of the CMS rats, and the level of Apo-A1 obviously decreased. Its contents in NE, DML-L, DML-M, and DML-H groups were significantly higher than those in the CMS model group. This result indicates that the total flavonoids from *D. moldavica* L. have protective effects. The pathology sections showed that the flavonoids have protective effects on the brain tissue of the CMS rats.

### 4.4. Antioxidative and Anti-Inflammatory Effects

Inflammatory response and oxidative stress are the key processes in the pathogenesis of CMS. In hypoxia tissue fluids, the levels of interleukins (ILs), such as IL-1*β*, IL-6, IL-18, and other inflammatory components, and the level of oxidation product malondialdehyde (MAD) significantly increase; in contrast, the contents of IL-10 and other anti-inflammatory factors, liver protein superoxide dismutase (SOD), glutathione catalase (GSH-Px), and other antioxidants significantly decrease. Our previous study showed different degrees of exudation, hyperemia, and inflammatory cell infiltration in the alveoli of CMS rats, demonstrating that hypoxia can activate and aggravate inflammatory immune response, reduce the anti-inflammatory ability of the body, aggravate the oxidative stress process, and reduce antioxidant ability [7, 21–23]. Apo-A1 has anti-inflammatory and antioxidative effects [24]. Apo-A1 plays an anti-inflammatory role by inhibiting the LPS-induced secretion of IL-1*β*, IL-6, and tumor necrosis factor-*a* (TNF-*α*) based on the interaction with megakaryocytes expressing ATP junction box transporter A1. When the content of Apo-A1 decreases, the release of these inflammatory cytokines is deregulated. This cycle leads to multiple organ dysfunction syndrome [25, 26]. Moreover, if the secretion of IL-1*β*, IL-6, TNF-*α*, and other inflammatory cytokines increases, the content of Apo-A1 in serum decreases. The decrease further causes the uncontrollable secretion of the inflammatory cytokines, thereby leading to multiple organ dysfunction syndrome. Our serological test results showed that the content of Apo-A1 in the CMS model group was downregulated, and Apo-A1 contents in the NE, DML-L, DML-M, and DML-H groups were obviously higher than those in the CMS model group. The effect in DML-H was better than that in the nifedipine group. Thus, the total flavonoids from *D. moldavica* L. have anti-inflammatory and antioxidative effects. In addition, we showed that the cerebral tissues of the CMS model rats had different degrees of meningeal vasodilation and hyperemia, cerebral parenchymal vasodilation, hemorrhage, and edema, accompanied by inflammatory cell infiltration and enlarged pyramidal cells. After gavage administration with the flavonoids, no inflammatory cell infiltration was observed in the brain tissue, hemorrhage and edema were alleviated, and the meninges and brain parenchyma and pyramidal cells had normal shapes. These results indicate that the flavonoids have a certain therapeutic effect on chronic hypoxia and brain hypoxia of CMS rats through anti-inflammatory effects.

The luteolin-7-O-*β*-D-glucuronide, apigenin-7-O-*β*-D-glucuronide, rosmarinic acid, and geraniol contained in the total flavonoids of *D. moldavica L* 7-O-*β*-D-glucuronide, serimarin, and robinin-7-O-*β*-D-glucuronide, all have good anti-inflammatory and antioxidant effects, so we speculate that the above-mentioned components in total flavonoids of *D. moldavica L* activate different signaling pathways to exert antioxidative and anti-inflammatory effects and finally produce certain therapeutic effects on CMS rats.

## 5. Conclusion

The results of this study showed that the total flavonoids of *D. moldavica L* can significantly reduce pulmonary artery pressure, improve brain tissue damage, and reduce serum Apo-A1 and Apo-E levels in CMS rats. The possible mechanisms may be that the total flavonoids of *D. moldavica L* improve energy metabolism, amino acid metabolism, protein metabolism, and antioxidative capacity in CMS rats.

## Figures and Tables

**Table 1:** Effects of total flavonoids from *D. moldavica* L. on pulmonary artery pressure （$\bar{x}$±*s*）.

Groups	*n*	Dosage （mg/kg）	PAP （mmHg）
CG	8	—	19.34 ± 1.32
MG	8	—	44.12 ± 3.25^*∗*^
NE	8	2.7	28.35 ± 2.24^*∗*^^#^
DML-H	8	400	26.12 ± 2.01^*∗*^^#▲^
DML-M	8	200	29.95 ± 3.22^*∗*^^#▲^
DML-L	8	100	33.12 ± 3.05^*∗*^^#^

*Note.* Compared with CG, ^*∗*^*P* < 0.05; compared with MG, #*P* < 0.05; compared with NE, ▲*P* < 0.05.

**Table 2:** Serum endogenous merabolite attribution and chemical shift.

No	Metabolite	Chemical shift (ppm)	Assignment
1	Lipid （LDL/VLDL)	0.85 (m), 1.25 (m), 2.74 (m)	C26 and C27, （CH_2_）n, C＝CCH2C＝C
2	Isoleucine	0.93 (t)， 1.00 (d)	*δ*-CH_3_, *β*-CH_3_
3	Leucine	0.97 (d)	*δ*-CH_3_
4	Valine	1.02 (d), 3.57 (d)	*γ*-CH3， *α*-CH2
5	3-Hydroxybutyrate	1.20 (d)， 2.31 (m)	*γ*-CH_3_， half*α*-CH_2_
6	Lactate	1.33 (d)， 4.11 (q)	CH_3_， CH
7	Alanine	1.46 (d)	CH_3_
8	Acetate	1.91 (s), 2.22 (s)	CH_3_
9	Glycoprotein	2.04 (s）, 2.14 (s)	NHCOCH_3_
10	Acetoacetate	2.27 (s)	CH_3_
11	Pyruvate	2.36 (s)	CH_3_
12	Glutamine	2.41 (m)	half*γ*-CH_3_
13	Citrate	2.52 (d), 2.66 (d)	Half-CH_2_
14	Creatine	3.03 (s), 3.93 (s)	CH_3_,CH_2_
15	Choline	3.21 (s)	N(CH3)O_3_
16	Taurine	3.42 (t)	CH_2_SO_3_
17	Proline	3.45 (m)	Half*δ*-CH_2_
18	Glycine	3.54 (s)	CH_2_
19	Citrulline	3.70 (m)	*α*-CH_2_
20	Alpha-glucose	3.54 (dd), 3.84 (m), 5.23 (d)	H2, half*δ*-CH2-C6, H1

^1^H-NMR metabolism multivariate statistical analysis of the sera of rats in the CMS model group.

**Table 3:** Changes in potential biomarkers in each group of rats.

No	Metabolite	Coefficient(r)					
			CG vs MG	MG vs NE	MG vs dml-l	MG vs dml-m	MG vs DML-H
		R^2^X	0.60	0.43	0.50	0.44	0.46
		Q^2^	0.90	0.73	0.75	0.67	0.71
1	Lipid （LDL/VLDL)		−0.91	0.71	0.73	0.79	0.75
2	Isoleucine		0.81	−0.81	−0.83	−0.73	−0.66
3	Leucine		0.91	−0.83	−0.87	−0.76	−0.74
4	Valine		0.94	−0.77	−0.84	−0.72	−0.77
5	3-Hydroxybutyrate		−0.86	0.81	0.73	0.73	0.80
6	Lactate		0.88	−0.82	−0.84	−0.71	−0.72
7	Alanine		0.82	−0.72	−0.91	−0.81	−0.80
8	Acetate		−0.79	−0.69	0.79	0.72	
9	Glcyprotein		0.76	−0.70	−0.71	−0.69	−0.68
10	Acetoacetate		0.83	−0.65	0.68	0.68	
11	Pyruvate		0.91	−0.77	−0.73	−0.77	−0.82
12	Glutamine		0.90	−0.69	−0.74	−0.69	−0.73
13	Citrate		0.85	−0.69	−0.70		
14	Creatine		0.82	−0.75	0.85	0.67	0.74
15	Choline			0.74	−0.86	−0.71	−0.64
16	Taurine		0.73	0.79	−0.88	−0.68	−0.63
17	Proline		0.69	0.71	−0.84	−0.64	−0.67
18	Glycine		0.89		−0.70	−0.52	−0.48
19	Citrulline		−0.58	0.80	0.81	0.70	0.70
20	Alpha-glucose		0.83	0.76	−0.87	−0.65	−0.72

**Table 4:** Pathway analysis results obtained with MetaboAnalyst

Key	Pathway name	Match status	Impact	-Log(p)
1	Glyoxylate and dicarboxylate metabolism	5/32	10.729	0.13757
2	Glycolysis/gluconeogenesis	4/26	8.6209	0.13034.
3	Alanine, aspartate, and glutamate metabolism	4/28	8.3214	0.11378
4	Glycine, serine, and threonine metabolism	4/34	7.5489	0.27117
5	Synthesis and degradation of ketone bodies	2/5	6.6329	0.6
6	Pyruvate metabolism	3/28	6.2624	0.26749
7	Arginine biosynthesis	2/14	4.4883	0.22843
8	Butanoate metabolism	2/15	4.3523	0.11111
9	Citrate cycle (TCA cycle)	2/20	3.7945	0.13672
10	Taurine and hypotaurine metabolism	1/8	2.3888	0.42857

**Table 5:** Effects of total flavonoids from *D. moldavica* L. on Apo-A1 and Apo-E contents in the sera of rats in each group （$\bar{x}$±*s*）.

Groups	N	Dosage (mg/kg)	Apo-E (ug/mL)	Apo-A1 (ug/mL)
CG	8	—	156.54 ± 4.61	584.21 ± 6.90
MG	8	—	102.78 ± 3.15^*∗*^	438.04 ± 7.80^*∗*^
NE	8	2.7	136.56 ± 5.03^*∗*^#	539.02 = 5.65^*∗*^#
DML.L	8	100	110.87 ± 3.78^*∗*^#	510.34 ± 2.41^*∗*^#
DML.M	8	200	129.22 ± 4.39^*∗*^#	522.29 ± 2.21^*∗*^#
DML.H	8	400	143.82 ± 2.33^*∗*^	563.20 ± 2.47^*∗*^

*Note.* Compared with CG, ^*∗*^*P* < 0.05; compared with MG, ^#^*P* < 0.05.

## Data Availability

The datasets used and/or analyzed during the current study are available from the corresponding author on reasonable request.
